# Motivation for Physical Activity as a Key Determinant of Sedentary Behavior Among Postsecondary Students

**DOI:** 10.1177/00469580241254032

**Published:** 2024-05-14

**Authors:** Rachel Surprenant, Isabelle Cabot, Caroline Fitzpatrick

**Affiliations:** 1 Cégep de Saint-Hyacinthe, Saint-Hyacinthe, QC, Canada; 2 Université de Sherbrooke, Sherbrooke, QC, Canada; 3 Cégep Édouard-Montpetit, Longueuil, QC, Canada

**Keywords:** motivation, screen time, physical activity, postsecondary students, sedentary behavior

## Abstract

It is known that the transition to adulthood represents a critical period of life when acquiring healthy behaviors can influence lifestyle and health throughout adulthood. Given the importance of the consequences of a sedentary lifestyle, identifying influence factors is key to improving healthy behaviors. The objective of this study is to explore the role of postsecondary students’ motivation toward physical activity in the association with their screen time and out-of-school physical activity practice. A total of 1522 postsecondary students (90% were aged 17-20 years) recruited from 17 postsecondary institutions completed the self-reported questionnaire during course time. Multivariate linear regression was used to assess the association between motivation to move including additional predictors of behavior such as intention and tendency to self-activate and self-reported screen time and physical activity controlling for age and sex. Motivation including all 3 motivational variables (interest, utility, competence) was negatively associated with screen time, *b* = −0.498 (95% CI between −0.635 and −0.361) and positively associated with moderate-to-vigorous physical activity, *b* = 133.986, (95% CI between 102.129 and 165.843). Of the 3 motivational variables, interest had the strongest negative association with screen time, *b* = −0.434 (95% CI between −0.551 and −0.317), and the strongest positive association with physical activity, *b* = 113.671, (95% CI between 86.396 and 140.946). These findings indicate that the motivation of postsecondary students toward physical activity significantly influences their behaviors, including screen time and physical activity engagement.

**What is already known on this topic?**
Some studies have linked adolescent screen time to physical activity, while others have found associations between motivation to be physically active and involvement in physical activity. However, less research has examined associations between motivation for physical activity and screen time.
**How does this research contribute to the field?**
This study contributes by exploring the relationship between motivation toward physical activity, considering three dimensions of motivation simultaneously, as well as intention to practice physical activity and tendency to self-activate, and screen time and physical activity practice among postsecondary students.
**What are this research’s implications toward practice?**
Our findings may help in the elaboration and implementation of healthy lifestyle interventions in school settings and suggest that teachers and practitioners direct their efforts on influencing student motivation and interest in physical activity.

## Introduction

The transition to adulthood is generally accompanied by a sharp drop in physical activity and fitness levels among students.^1-7^ For example, according to one study, 37.2% of postsecondary students (n = 1886) engaged in less than 10 min of weekly physical activity outside of school.^8^ At the same time, the amount of time allocated to recreational screen use among young adults is considerable, averaging 4.7 h per day.^9^ Consequently, it is unsurprising that a significant number of postsecondary students do not meet 24-Hour movement guidelines for adults.^10^ More specifically, 38.9% fail to achieve the recommended 150 min per week of moderate-to-vigorous physical activity, 63.8% exceed the maximum of 3 h per day of recreational screen time, and 43.7% exceed the recommended limit of 8 h of sedentary time per day.^9^

Screen time and physical activity behaviors adopted during the transition to adulthood may become important predictors of long-term health, due to the predisposition to carry such behavioral patterns into adulthood.^11^ Both are also independently associated with health status among youth.^12-14^ In addition, a sedentary lifestyle, including high levels of screen time and low levels of physical activity, is considered one of the world’s leading causes of mortality,^15^ and is linked to cardiovascular disease,^16,17^ type 2 diabetes,^18^ and certain types of cancers.^19,20^ Sedentary behaviors are also associated with higher risks of anxiety,^21^ depression,^22^ and indicators of psychological distress such as low self-esteem, feelings of loneliness, and high levels of stress.^23^ In addition, screen media use in particular is associated with increased levels of anxiety^24^ and depression^25^ in youth.

From a public health perspective, it is essential to identify the modifiable factors or determinants of sedentary behaviors in postsecondary students in order to improve interventions and health promotion efforts. Youth motivation is an essential determinant of their eventual engagement in a behavior.^26,27^ Motivation helps trigger engagement in behavior, which translates into participation in a task.^28^ Consequently, the concept of engagement is closely linked to that of motivation, since motivation precedes engagement.^29,30^ We understand motivation as “a process in which goal-directed activity is instigated and sustained” (p. 5).^31^ Various models of motivation (including Expectation-Value, Self-determination and Motivational dynamics models) from different approaches agree that motivation includes specific dimensions of interest (eg,: I enjoy being physically active), utility (eg,: Physical activity practice is useful for me), and perceived competence (eg,: When I am physically active, I feel competent).^30,32-36^

In socio-cognitive approach theories, interest consists of emotions and cognitions and is divided into 2 types of interest: situational and personal. Situational interest is mainly emotional, temporary and dependent on the environment, while personal interest content emotions and cognitions, is stable and inherent to the individual. In the process of developing an interest, situational interest precedes the deployment of personal interest.^37^ Interest is similar of intrinsic motivation in self-determination theory.^38^ For its part, the utility attributed to an activity is defined as the person’s assessment of the compatibility between this activity and the person’s goal pursuit.^34,39^ It’s similar to extrinsic motivation in self-determination theory.^40^ Competence refers to an individual’s perception of their ability to achieve an activity properly.^30^ In self-determination theory, it represents 1 of the 3 fundamental psychological needs.^41^

**Figure 1.**
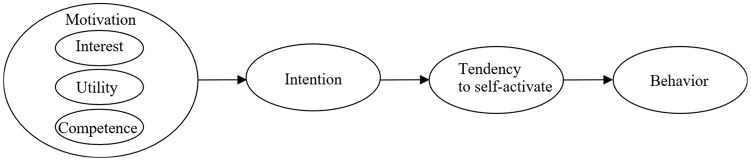
Relationship between motivation, intention, tendency to self-activate and behavior.

Motivation for physical activity in turn influences predictors of behavior as the intention to engage in practicing physical activity and the tendency to self-activate. Indeed, intention is considered closer to behavior than motivation because it includes the planning stage of the intended behavior.^42^ Conceptually, intention is between motivation and the concrete engagement in the planned behavior.^43^ The tendency to self-activate, reflecting a personal propensity to engage in what has been planned, is closely and positively linked to motivation and this has been examined in young adults.^44^ For example, as illustrated in Figure 1, a person might be motivated by an activity (eg, cycling), and plan a time to engage in it (intention). However, just before the planned activity (cycling), an alternative motivational activity might arise (eg, playing a video game with friends), creating a motivational conflict^45,46^ that threatens the execution of the planned behavior. If the person possesses a tendency to self-activate (meaning a predisposition to carry out what is planed, as defined earlier), he or she would be more likely to enact the planed behavior. Although tendency to self-activate has been linked to motivation to move, less is known about its potential role as a determinant of screen time in young adulthood.

A literature review was conducted that examines how motivation toward physical activity can influence both screen time and the practice of physical activity. Some studies have linked adolescent screen time to moderate-to-vigorous physical activity.^47-49^ Others have found associations between motivation for physical activity, and engagement in physical activity in adolescents.^50,51^ To date, less research has examined associations between motivation for physical activity and screen time. This research gap contributes to the pertinence of this study. The objective of this study is to examine how motivation for physical activity contributes to screen time and physical activity practice among young adults. More specifically we examine how motivation and its dimensions (interest, utility, competence) and additional predictors of behavior (intention and tendency to self-activate) are associated with screen time and physical activity. We hypothesize that young adult’s motivation toward physical activity will be associated with lower screen time and greater physical activity levels.

## Methods

### Participants

In the present study, we use a community-based convenience sample of 1706 participants between the ages of 17 and 42 recruited from 17 colleges in the province of Quebec, Canada called “collèges d’enseignement général et professionnel” (CEGEP). CEGEPs are publicly funded postsecondary educational institutions, offering 2-year pre-university programs and 3-year vocational programs. A cohort of 815 students was recruited during the Fall 2021 semester and a second cohort of 891 students was recruited during the Winter 2022 semester. Participants provided informed consent and completed a questionnaire during their physical education class. This study received approval from the ethics review boards of all participating institutions, and all participants signed a written informed consent before completing the survey. Students with missing data on continuous or control variables were excluded from the study (10.8% of the baseline sample), resulting in a final sample of 1522 students. In total, there were 923 females (60.6%) and 595 males (39.1%) participants.

## Measures

### Predictors

#### Motivation

We created a variable to measure motivation for physical activity, comprising 14 items from 3 scales, each targeting a dimension of motivation; 6 items measure interest in physical activity; 4 items measure the utility of physical activity; and 4 items measure competence in physical activity practice. Participants rated each item on a Likert scale ranging from 1 (strongly disagree) to 5 (strongly agree). We used the mean of each scale (interest, usefulness, competence) to represent this variable ranged from 1 to 5.

### Dimensions of Motivation

#### Interest

This variable derives from items developed and validated in the studies by Cabot et al,^27,52^ and was subsequently adapted for a study focusing specifically on interest in physical activity.^53^ This variable contains 6 items (λ = 0.90) and represents the two dimensions of personal interest (cognitive and affective). The cognitive dimension enables the participant to express interest in learning about physical activity (eg,: I enjoy learning about physical activity even outside the school context); the affective dimension enables emotional expression of interest in physical activity (eg,: I always want to be physically active).

#### Utility

This variable comprises 4 items (λ = 0.85) derived from the utility attributed to the practice of physical activity scale: It’s important for me to engage in regular physical activity practice; I find it worthwhile to engage in regular physical activity practice; Regular physical activity practice is useful for me; and Regular physical activity practice brings me benefits in life.^54^ Studies of Hulleman and Harackiewicz^34^ justified a slight adjustment of the items by reformulating them in a more personal way. For example, the item “Regular physical activity practice is useful”^54^ became “Regular physical activity practice is useful for me.”^53^

#### Competence

This variable represents the participants’ perceived competence toward the practice of physical activity^54^ which is composed of 4 Likert-type items (λ = 0.84): I am good at physical activity; When I do physical activity, I am among the best; When I do physical activity, I feel competent; and I know many things about physical activity.

#### Intention

This variable reflects the intention to meet physical activity guidelines and was measured using the following question: “The World Health Organization recommends at least 150 min of moderate-intensity physical activity or at least 75 min of vigorous-intensity physical activity each week. Over the next 3 months, do you intend to follow these recommendations?” Participants rated this question as follows: (1) yes; (2) yes, maybe; (3) no, probably not; (4) no. Responses were reverse coded so that higher scores reflect higher levels of intention to practice physical activity.

#### Tendency to self-activate

The tendency to be active relates specifically to an individual’s ability to maintain control over planned behavior until it is realized. This variable was inspired by the notion of behavioral control and was developed from validated items.^44^ The scale comprises 4 items (λ = 0.85) on a 5-point Likert scale (1-5) from strongly disagree to strongly agree: When I want to do physical activity, I do it; I am able to put myself in action to influence my physical condition; When I plan to practice physical activity, I really do it; I am able to go beyond my desire to be active: I really am).

### Dependent Variables

#### Screen time

Screen time was assessed using the following question: How many hours a day do you usually spend on screen during your free time (outside school or work)? To ensure the participant excluded screen time devoted to school or work obligations, an additional question specifically targeted work-based screen time.

#### Physical activity

Participants reported physical activity practice during a typical week over the last 3 months before the beginning of the semester. Specifically, they indicated the duration (minutes/week), nature (eg, swimming, jogging, playing soccer), and intensity (eg, low, moderate, vigorous) of physical activity (Table 1,^55^). The responses were then used to estimate a single variable reflecting total weekly minutes of moderate and vigorous-intensity physical activity. The number of minutes spent in vigorous-intensity activity was multiplied by 2 and then added to the number of minutes spent in moderate-intensity activity to reflect the WHO’s recommendation in which the duration of vigorous-intensity activity is equivalent to twice the duration of moderate-intensity activity. The World Health Organization^56^ recommends a minimum of 150 min of moderate-intensity endurance activity or a minimum of 75 min of vigorous-intensity endurance activity per week for health benefits.

**Table 1.** Descriptive Characteristics.

	Total sample (n = 1522)
Sex, n (%)	
Male	595 (39.10)
Female	923 (60.60)
Other/Preferred not to answer	4 (0.30)
Age, years, n (%)	
17-18	704 (46.20)
19-20	667 (43.80)
≥21	151 (10.00)
Motivation, (mean, SD)	
Motivation (include interest, utility, competence)	3.57 (0.86)
Interest	3.35 (1.00)
Utility	4.03 (0.89)
Competence	3.33 (0.95)
Intention, (mean, SD)	3.28 (0.77)
Tendency to self-activate, (mean, SD)	3.53 (0.99)
Screen time (hours/day, mean, SD)	3.87 (2.35)
Physical activity (minutes/week, mean, SD)	380.74 (512.27)

*Note.* SD = standard deviation.

#### Covariates

Participants reported age (in years) and sex, as either male or female.

### Data Analysis

We estimate a series of multiple linear regression with 95% confidence intervals to examine the contribution of young adult’s motivation for physical activity to their screen time and physical activity practice, adjusting for age and sex. The intention to engage in physical activity practice and the tendency to self-activate were also included as additional predictors in the model examining the associations between motivation and screen time and physical practice. To limit the impact of extreme values, values outside ± 3 standard deviation thresholds were considered outliers and removed from the analyses.^57^ All analyses were conducted using IBM SPSS Statistics for Windows, Version 28.0 (IBM Corp., Armonk, NY, USA).^58^

## Results

Descriptive statistics (n, % or mean, SD) are presented in Table 1 for the total sample. A total of 1522 participants (89.2% of the baseline sample) provided complete data and were used for the analysis. Our sample was predominantly female (60.6% females). Participants were aged 17 to 42 years (*M* = 19.09, SD = 2.18) but 90.0% were between age 17 and 20 years. Participants in our sample spent on average 3.87 h per day for recreational screen time and 48.2% of the sample exceeded daily recommendations of 3 h or less per day. With regard to physical activity, participants were active an average of 381 min per week and 43.8% of the sample did not meet physical activity guidelines.

### Regression Results

Table 2 presents the results of the multivariate analyses used to model the association between motivation toward physical activity, including intention or tendency to self-activate and screen time and physical activity. Significant associations were observed between all motivational variables (interest, utility, competence) and screen time. Of all three dimensions for motivation to move, interest had the strongest negative association with screen time, *b* = −0.434 (95% CI between −0.551 and −0.317). Each unit of utility attributed to physical activity made significant negative contributions to screen time *b* = −0.360 (95% CI between −0.492 and −0.227). Finally, competence in physical activity practice scale was also associated with significant decreases in screen time (hours), *b* = −0.426 (95% CI between −0.552 and −0.300). Model Motivation presents unstandardized regression coefficients for motivation including all 3 motivational variables (interest, utility, competence), and adjusted for age and sex. In this Model, we observed the strongest negative association with screen time, *b* = −0.498 (95% CI between −0.635 and −0.361). In Model Intention, the intention to practice physical activity was included in the Model Motivation. Significant associations were observed with screen time, *b* = −0.384 (95% CI between −0.551 and −0.218) after controlling for covariates. Instead of intention, the tendency to self-activate was included in the last model which made smaller contributions to screen time, *b* = −0.326 (95% CI between −0.561 and −0.091).

**Table 2.** Unstandardized regression coefficients and 95% confidence intervals (CIs) for screen time and physical activity according to motivation for physical activity (n = 1522).

	Screen time B (95% CI)	Physical activity B (95% CI)
Motivational variables		
Interest	−0.434 (−0.551, −0.317)^**^	113.671 (86.396, 140.946)^**^
Utility	−0.360 (−0.492, −0.227)^**^	103.421 (72.717, 134.125)^**^
Competence	−0.426 (−0.552, −0.300)^**^	112.137 (82.827, 141.447)^**^
Model motivation	−0.498 (−0.635, −0.361)^**^	133.986 (102.129, 165.843)^**^
Model intention	−0.384 (−0.551, −0.218)^**^	117.923 (79.310, 156.536)^**^
Model tendency to self-activate	−0.326 (−0.561, −0.091)^*^	95.543 (40.497, 150.589)^**^

*Note.* Screen time variable is measured in hours per day. Physical activity variable is measured in minutes per week. Motivation includes interest, utility, competence, and is adjusted for age and sex. Model Intention is Motivation plus adjustment for intention. Model Tendency to self-activate is Motivation plus adjustment for Tendency to self-activate.

* *P* < .01. ^**^P < .001.

Significant associations were also observed between all motivational variables and physical activity practice. The dimension of interest had the strongest positive association with physical activity, *b* = 113.671, (95% CI between 86.396 and 140.946). Each unit of utility attributed to physical activity made significant positive contributions to physical activity, *b* = 103.421 (95% CI between 72.717 and 134.125). Competence in physical activity practice was also associated with significant increases in physical activity (minutes), *b* = 112.137, (95% CI between 82.827 and 141.447). Model Motivation made the strongest positive association with physical activity, *b* = 133.986, (95% CI between 102.129 and 165.843). In Model Intention, being motivated by physical activity was associated with an increase of *b* = 117.923 (95% CI between 79.310 and 156.536) in the physical activity practice scores after controlling for covariates. Finally, Model Tendency to self-activate made smaller contributions to physical activity, *b* = 95.543 (95% CI between 40.497 and 150.589).

## Discussion

As far as we know, the present study is the first to describe the relationship between motivation toward physical activity, screen time and physical activity practice among postsecondary students. In addition, this is the first study to simultaneously consider these dimensions of motivation as well as intention to practice physical activity and tendency to self-activate. After adjusting for age and sex, we found that motivation for physical activity was the strongest predictor of youth screen time and moderate-to-vigorous physical activity. Being interested in and feeling competent in performing physical activity were the two dimensions of motivation most strongly linked to physical activity involvement and time spent using screens in our sample of young adults. Neither intention to engage in physical activity nor tendency to self-activate were related to our outcomes. This is consistent with another study that demonstrates the key role of intrinsic motivation, which is conceptually linked to interest in physical activity, in enhancing levels of physical activity.^59^

Our study extends previous work on adolescents by suggesting that similar motivational processes may be involved in the adoption of lifestyle habits.^51^ The present result is also in line with a recent study of motivation for physical activity and sedentary behaviors among secondary students which revealed that intrinsic motivation was negatively linked with sedentary behaviors and positively associated with physical activity engagement.^60^ Furthermore, our findings align with another study showing positive associations between healthy behaviors (eg, physical activity and healthy weight control behaviors) and autonomous motivation.^61^

Some limitations should be considered. First, our study used a cross-sectional design that does not allow us to infer the directionality in the observed associations. For instance, it may be case that accumulating lower levels of physical activity and spending more time in front of screens are contributing to decrease motivation in youth. Experimental or longitudinal studies could examine changes in the relationship over time and clarify the direction of the association. In addition, we used self-reported measures of motivation, screen time, and physical activity involvement. This could have resulted in shared measurement error or social desirability bias. Indeed, self-reported measures, particularly reports of moderate-to-vigorous physical activity, can lead to overestimations.^62^ As such, future studies using objective measures, such as accelerometers are warranted. Another limitation is the use of a convenience sample which is potentially limiting the generalizability of the findings. Finally, we had limited socioeconomic variables for the participants. For instance, the socioeconomic status or ethnicity of the students may moderate the observed associations.

The main strength of this study is our ability to simultaneously consider the impact of multiple dimensions of motivation on screen time and physical activity in young adults. To date, most research has focused on the role of the sociodemographic characteristics of individuals in the adoption of lifestyle choices.^49,63^ Though important, these determinants are likely to be more difficult to leverage through interventions. Our study is one of the first to examine how psychological motivation for physical activity contributes to the adoption of healthy behaviors. Furthermore, our study provides a detailed account of these associations by considering multiple dimensions of motivation (ie, interest, utility, competence, intention, and tendency to self-activate).

The present study may help the elaboration and implementation of healthy lifestyle interventions in the postsecondary school setting. First, our results indicate the importance of psychological interventions aimed at encouraging students to adopt or maintain a healthy, active lifestyle which are consistent with recent work showing that pedagogical practices focusing on enjoyment and perceived benefits of physical activity influence students to get moving.^64,65^ Feeling pleasure is one of the important factors that makes people do more physical activity.^66^ Our findings suggest that teachers and practitioners should work specifically to influence motivation and improve interest in physical activity by setting up activities that bring pleasure and positive emotions. Moreover, focusing on specific motivational behaviors to provide necessary support could improve healthy behaviors.^67^

## Conclusion

Motivation to move plays an important role in screen time and the practice of physical activity outside of the school context among postsecondary students. Future studies should use longitudinal designs and objective measures of physical activity to better understand the association between students’ motivation, screen time and physical activity practice. Our results support the development of interventions to target the motivation to move in physical education classes to reduce sedentary behavior among students.
